# Longer-term virologic outcomes on tenofovir-lamivudine-dolutegravir in second-line ART

**DOI:** 10.4102/sajhivmed.v26i1.1677

**Published:** 2025-04-30

**Authors:** Jennifer K. van Heerden, Ying Zhao, Claire M. Keene, Rulan Griesel, Zaayid Omar, René Goliath, Kayla Delaney, Gert van Zyl, Gary Maartens, Graeme Meintjes

**Affiliations:** 1 Wellcome Centre for Infectious Diseases Research in Africa, Institute of Infectious Disease and Molecular Medicine, University of Cape Town, Cape Town, South Africa; 2 Department of Biochemistry, Medical Sciences Division, University of Oxford, Oxford, United Kingdom; 3 Department of Medicine, Faculty of Health Sciences, University of Cape Town and Groote Schuur Hospital, Cape Town, South Africa; 4 Médecins Sans Frontières, Cape Town, South Africa; 5 Health Systems Collaborative, NDM Centre for Global Health Research, Nuffield Department of Medicine, University of Oxford, Oxford, United Kingdom; 6 Division of Clinical Pharmacology, Department of Medicine, University of Cape Town and Groote Schuur Hospital, Cape Town, South Africa; 7 Division of Medical Virology, Faculty of Medicine and Health Sciences, Stellenbosch University and Tygerberg Hospital, Cape Town, South Africa; 8 Blizard Institute, Faculty of Medicine and Dentistry, Queen Mary University of London, London, United Kingdom

**Keywords:** HIV, tenofovir-lamivudine-dolutegravir, dolutegravir, drug resistance, second-line, South Africa

## Abstract

**Background:**

Dolutegravir in second-line antiretroviral therapy (ART) is more effective with recycled tenofovir than switching to zidovudine. However, dolutegravir resistance is more frequent in second-line compared to first-line ART.

**Objectives:**

We report long-term virologic outcomes from a clinical trial.

**Method:**

AntiRetroviral Therapy In Second-line: investigating Tenofovir-lamivudine-dolutegravir (ARTIST) was a randomised, double-blind, phase II clinical trial. Eligible participants had two consecutive HIV-1 RNA ≥ 1000 copies/mL on first-line ART, mostly tenofovir-emtricitabine-efavirenz. Participants were switched to tenofovir-lamivudine-dolutegravir (TLD) with lead-in 50 mg dolutegravir twice daily in stage one (*n* = 62), and randomised to TLD with additional lead-in 50 mg dolutegravir or placebo for the first 14 days in stage two (*n* = 130). We present results up to 158 weeks, combining stages one and two.

**Results:**

We enrolled 192 participants: 127/176 (72%) had resistance (Stanford score ≥ 15) to both tenofovir and lamivudine. At week 48, 151/186 (81%; 95% confidence interval [CI] 75%, 87%) had HIV-1 RNA < 50 copies/mL. Of 127 participants with follow-up through week 158, 78% (95% CI 70%, 85%) maintained HIV-1 RNA < 50 copies/mL, 11% had HIV-1 RNA 50–999 copies/mL, and 11% had HIV-1 RNA ≥ 1000 copies/mL. Twenty-nine participants met criteria for resistance testing: one developed intermediate-level dolutegravir resistance (G118R mutation) at week 96, and one had high-level dolutegravir resistance (E138K, G118R, G163R, T66A mutations) detected at week 146.

**Conclusion:**

Among adults switching to TLD with detectable HIV-1 RNA and substantial tenofovir and lamivudine resistance, a high proportion maintained virologic suppression up to 158 weeks. Emergent dolutegravir resistance occurred in ~1% of participants after 2–3 years on second-line TLD.

**What this study adds:** This study reports longer-term outcomes of second-line TLD from a South African clinical trial cohort. Virologic suppression was maintained in approximately 80%, and dolutegravir resistance was uncommon. These data support the use of TLD in second-line regimens without prior resistance testing.

## Introduction

Dolutegravir, a second-generation integrase strand transfer inhibitor (INSTI), currently forms part of the WHO-recommended first-line and second-line antiretroviral therapy (ART) regimens.^1^ As genotypic drug-resistance testing is not readily accessible at the time of second-line initiation in most low- and middle-income countries (LMICs), substituting tenofovir with zidovudine has previously been recommended to ensure at least one fully active nucleoside reverse transcriptase inhibitor (NRTI).^1,2^ However, more recently, second-line ART has been shown to be more effective and better tolerated with recycled tenofovir than switching to zidovudine.^3^ As a result, tenofovir-lamivudine-dolutegravir (TLD) taken as a fixed-dose combination is the preferred second-line option, and is initiated without the requirement of resistance testing prior to switching regimens in programmatic settings.^4,5^

Concern has been expressed that dolutegravir resistance may be selected among patients switching to dolutegravir with no fully active NRTI.^6,7^ Emergent dolutegravir resistance is rare, but has been reported in a small proportion of patients switching to second-line dolutegravir-based regimens in large randomised trials and in programmatic settings.^3,6,8,9^ In the Nucleosides and Darunavir/Dolutegravir in Africa (NADIA) Trial, 9 of 235 participants (4%) developed dolutegravir resistance mutations by 96 weeks, three of which occurred in the TLD group.^3^ In a prospective observational study conducted in Malawi of adults on first-line ART switching to TLD, 2 of 101 participants (2%) who were viraemic at switch developed dolutegravir resistance at 6 months.^8^ As TLD is widely used as second-line ART in LMICs, substantial emergence of dolutegravir resistance would have major public health implications.^4,5,10^

Long-term follow-up of patients on TLD as second-line therapy is important; first, to assess the longer-term rates of virologic suppression and thus the durability of TLD, and, second, to determine the incidence of and risk factors for emergent dolutegravir resistance. Here, we describe the longer-term virologic outcomes of participants from the AntiRetroviral Therapy In Second-line: investigating Tenofovir-lamivudine-dolutegravir (ARTIST) trial, a South African cohort on TLD as second-line therapy, with up to 158 weeks of follow-up.

## Research methods and design

### Study design, participants and setting

In this report, we present prospective data of the longer-term (≥ 48 weeks) virologic outcomes of participants from the ARTIST trial. This cohort has been described in detail elsewhere.^11,12,13^ In brief, ARTIST was a two-stage interventional trial conducted from July 2019 to October 2022. We enrolled ART-experienced, INSTI-naïve adult participants (≥ 18 years old) who experienced virologic failure (defined as two consecutive HIV-1 RNA ≥ 1000 copies/mL taken 2–24 months apart) on a first-line non-nucleoside reverse transcriptase inhibitor (NNRTI)-based ART regimen.^11,13,14^

In stage one of ARTIST, all participants were initiated on TLD as second-line ART with an additional lead-in 50 mg dose of dolutegravir daily for the first 14 days to overcome the inducing effect of efavirenz.^13^ In stage two we conducted a non-comparative, double-blind, randomised phase II trial and participants were randomised to receive either an additional lead-in dose of dolutegravir 50 mg daily or placebo for the first 14 days with TLD.^11,14^

Participants from both stages of the ARTIST trial were followed up at the research study site with regular HIV-1 RNA sampling (intervals outlined below) for a minimum of 48 weeks and until completion of ARTIST stage two in October 2022 (hereafter referred to as the clinical trial period). After this, participants were referred back to their local HIV clinic for continuation of TLD and routine HIV care. From May to September 2023, an attempt to contact all surviving ARTIST participants was made, and, if contactable and available, one to three post-trial visits were scheduled (hereafter referred to as the post-trial period).

All visits were conducted in Khayelitsha, Cape Town, South Africa, a large, peri-urban informal settlement where approximately 500 000 people reside.^15^ Participants originated from three primary care clinics: Site B Community Health Centre (CHC), Michael Mapongwana CHC, and Site C CHC.

### Procedures

Detailed procedures and protocols for ARTIST stage one and stage two have been published elsewhere.^13,14^ HIV-1 RNA samples were collected at the start of the study, weeks 4, 8, 12, 16, 20, 24, 36, and 48. For those reaching later time points within the clinical trial period, HIV-1 RNA was sampled at weeks 72, 96, 120, and 144. A ±14-day time window (±16 days in stage two) around visits was used until week 20 and a ±6-week time window from the week 24 visit. During the post-trial period, all contactable and willing participants had at least one visit with HIV-1 RNA sampling, with all visits being conducted at time points greater than 72 weeks after initiation of TLD. In participants with HIV-1 RNA values ≥ 50 copies/mL, the HIV-1 RNA was repeated 28 days later (±16-day time window). All participants with HIV-1 RNA ≥ 50 copies/mL received enhanced adherence counselling in their home language during the trial and post-trial period. HIV-1 RNAs were measured using Abbott Realtime® HIV-1 polymerase chain reaction assay (Abbott Molecular, Des Plaines, Illinois, United States), which quantifies virus RNA over a range of 20 to 10^7^ copies/mL.

Genotypic antiretroviral resistance testing (GART) using Sanger sequencing was performed retrospectively for all participants on samples collected at study entry. During the clinical trial period, if any HIV-1 RNA after week 12 was ≥ 50 copies/mL, or if there was < 1 log_10_ decline in HIV-1 RNA from the start of the study, or if HIV-1 RNA was suppressed and subsequently rebounded to ≥ 50 copies/mL, enhanced adherence counselling was performed, and HIV-RNA was repeated after 2 weeks. Participants with a repeat HIV-1 RNA ≥ 500 copies/mL had plasma sent for GART. At post-trial follow-up, all participants with HIV-1 RNA ≥ 500 copies/mL had plasma sent for GART unless they reported having interrupted ART at the time of follow-up, in which case they were re-initiated on therapy and GART only performed if the repeat HIV-1 RNA was ≥ 500 copies/mL after 28 days on TLD. GART was performed at the National Health Laboratory Service Virology Laboratory at Tygerberg Hospital, Cape Town, South Africa. Amplification of the *pol* gene containing reverse transcriptase, protease, and integrase regions was performed using the ThermoFisher real-time PCR assay (ThermoFisher Scientific Inc., Waltham, Massachusetts, United States). We used the Stanford HIV drug-resistance database (HIVdb algorithm, version 8.9 for stage one, version 9.1 for stage two, and version 9.5 for post-trial follow-up) to determine HIV-1 drug-resistance mutations (DRMs) and drug-susceptibility interpretations. Resistance was classified with the Stanford algorithm, with a score of ≥ 15 indicating at least low-level resistance.

We sampled tenofovir diphosphate (TFV-DP) concentrations in dried blood spots at week 48 in all participants, and during the post-trial follow-up period in participants with HIV-1 RNA ≥ 50 copies/mL. For TFV-DP dried blood spot samples, we collected ethylenediaminetetraacetic acid blood samples, then 50 µL of whole blood was pipetted onto Whatman™ 903 Proteinsaver cards (Whatman™ [Cytiva], Buckinghamshire, United Kingdom) which were dried overnight and then stored in airtight freezer-safe bags at –80 °C. A validated indirect method for quantifying TFV-DP was used by the laboratory of the Division of Clinical Pharmacology, University of Cape Town, which has been described elsewhere.^16^ The assay has a lower limit of quantification of 16.6 fmol/punch.

### Outcomes

We previously reported the primary outcomes for stage one and stage two of ARTIST.^11,13^ Using a modified intention to treat (mITT) analysis and the Food and Drug Administration snapshot algorithm, we reported the proportion of participants who had a plasma HIV-1 RNA < 50 copies/mL at week 24.^13,14^ The similar rates of virologic suppression at this time point in both stages formed the basis of our rationale to combine the data sets for further analyses.

Here, we combine data from stage one and stage two to report the longer-term (≥ 48 weeks) virologic outcomes at visits conducted within the clinical trial period up to week 144 as well as in the post-trial period, with follow-up up to 158 weeks. Specifically, we report the combined (stage one and stage two) cohort week 48 results, reporting the proportions of participants with available data who had a plasma HIV-1 RNA value < 50 copies/mL, the proportion with low-level viraemia (50 copies/mL – 999 copies/mL), and the proportion with HIV-1 RNA ≥ 1000 copies/mL, at specified durations on TLD.

We compare the week 48 with the week 96 virologic outcomes of participants on second-line TLD, and describe transitions between virologic suppression categories. To assess longer-term outcomes outside of the clinical trial setting, we compared the week 48 and post-trial follow-up virologic outcomes, and describe transitions between virologic suppression categories. In addition, we analysed the cohort who was virologically suppressed at week 48 to report the proportion of participants with subsequent rebound viraemia (defined as HIV-1 RNA ≥ 50 copies/mL after achieving virologic suppression), and the proportion of these participants who resuppressed after enhanced adherence counselling.

We did stratified analyses of the primary outcome, stratifying virologic outcomes by the presence or absence of NRTI resistance to both lamivudine and tenofovir at enrolment. To account for missing data at later time points, we combined HIV-1 RNA data from the clinical trial period, the post-trial follow-up visits and additional samples taken by local antiretroviral clinics (HIV-1 RNAs quantified using the same laboratory method as described above). We present these data separately.

In addition, we report the proportion of participants with emergent INSTI resistance mutations detected on GART. We describe the emergent resistance mutations, dolutegravir resistance level, HIV-1 RNA trajectories and profiles in these participants.

Secondary outcomes for this observational report included assessing the longer-term clinical outcomes in participants on TLD as second-line ART. We report the clinical outcomes (including all-cause mortality, hospitalisation, and incident tuberculosis) and objective adherence measures (quantified using TFV-DP concentrations in dried blood spots) in participants who were contactable and attended post-trial follow-up.

### Statistical analysis

Appropriate packages from R software version 4.2 (R foundation for Statistical Computing, Vienna, Austria) and STATA software version 17 (STATA Corporation, College Station, Texas, United States) were used for statistical analysis. Alluvial diagrams depicting virologic outcomes at different time points were drawn using *alluvial* (version 1.2) and *ggalluvial* (version 0.12.5) packages.^17,18^ We describe categorical data using proportions with 95% confidence intervals (CIs), and used the binomial exact method to calculate all 95% CIs. Median values (with interquartile ranges [IQR]) for non-parametric data or mean values (with standard deviations) for parametric data were used to describe continuous data. Between-group differences were analysed using chi-squared tests (or Fisher’s exact tests if the number in any cell was ≤ 5) for categorical data and Wilcoxon Rank Sum tests for non-parametric continuous data.

### Ethical considerations

Ethical approval was granted by the Human Research Ethics Committee at the University of Cape Town (reference numbers: 039/2019 and 288/2023). All participants provided written informed consent for inclusion in ARTIST and a separate informed consent for post-trial follow-up was obtained from ARTIST participants at the first post-trial visit. Participant data confidentiality was maintained throughout the study. This study was registered on ClinicalTrials.gov (reference number: NCT03991013).

## Results

### Participants and characteristics at enrolment

**FIGURE 1:**
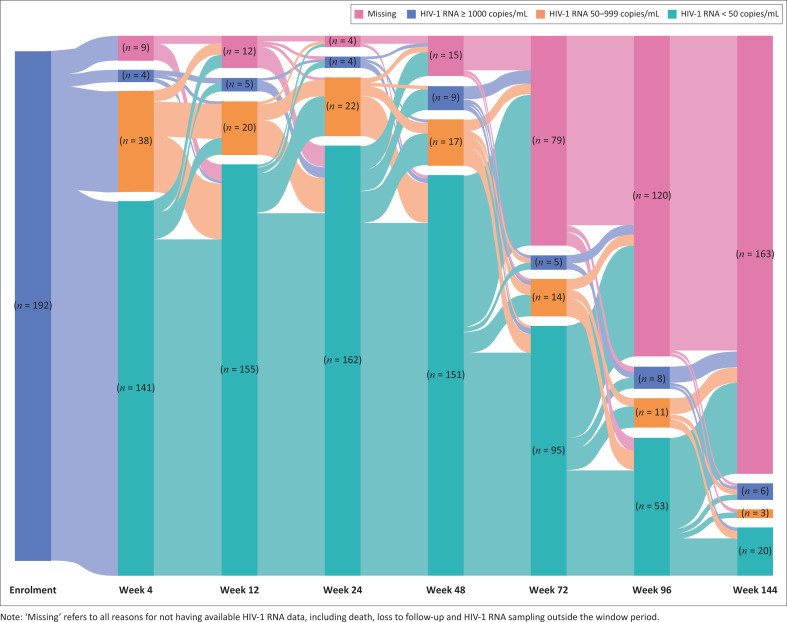
Virologic outcomes for participants in the ARTIST study at specified follow-up visits during the clinical trial period.

We enrolled 192 participants in ARTIST, 62 participants during stage one and 130 in stage two. The duration of longer-term follow-up and number of participants with available HIV-1 RNA results at each visit are shown in the Online Appendix 1, Figure 1-A1. HIV-1 RNA results were available in 177 participants at the week 48 visit, 72 participants at the week 96 visit during the clinical trial period, and 127 participants were contactable and attended post-trial follow-up (Online Appendix 1, Figure 1-A1). Participants at the post-trial visit had a median duration of 126 weeks (IQR 101, 162) since the initiation of TLD as second-line ART, with 32/127 (25%) having completed ≥ 158 weeks on TLD as second-line ART.

The baseline characteristics (i.e., at initial enrolment into ARTIST) for all participants and for those included in the week 96 and post-trial follow-up analyses are shown in Table 1. Of the 192 participants, 191 were previously on tenofovir-emtricitabine-efavirenz as first-line ART and 26/192 (14%) had previous exposure to stavudine or zidovudine. GART results at enrolment into ARTIST were available in 176/192 (92%) participants. The K65R mutation was detected in 76/176 (43%) participants and the M184V/I mutation in 154/176 (88%), with other NRTI mutations described in the Online Appendix 1, Table 1-A1. In 127/176 (72%) participants, at least low-level resistance (Stanford score ≥ 15) to both tenofovir and lamivudine was present at enrolment into the trial and prior to initiation of TLD.

**TABLE 1:** Characteristics of participants in the ARTIST cohort at the time of initiation of tenofovir-lamivudine-dolutegravir as second-line antiretroviral therapy.†

Characteristic	All participants in the ARTIST cohort ‡ (*N* = 192)					Participants included in the week 96 analysis (*N* = 72)					Participants included in the post-trial follow-up analysis (*N* = 127)				
	*n*	*N*	%	Median	Interquartile range	*n*	*N*	%	Median	Interquartile range	*n*	*N*	%	Median	Interquartile range
**Age**	-	-	-	38	33–45	-	-	-	38	31–45	-	-	-	39	33–46
**Sex**															
Female	132	192	69	-	-	56	72	78	-	-	90	127	71	-	-
Male	60	192	31	-	-	16	72	22	-	-	37	127	29	-	-
**BMI §**	-	-	-	28.6	23.4–34.3	-	-	-	29.1	23.4–34.7	-	-	-	30.7	23.7–35.2
Underweight	7	192	3.6	-	-	3	72	4	-	-	2	127	1.6	-	-
Normal	58	192	30	-	-	19	72	26	-	-	35	127	28	-	-
Overweight	41	192	21	-	-	17	72	24	-	-	21	127	17	-	-
Obese	86	192	45	-	-	33	72	46	-	-	69	127	54	-	-
**CD4 lymphocyte count at enrolment (cells/µL)**	-	-	-	250	170–344	-	-	-	277	190–379	-	-	-	246	175–339
**Log_10_ HIV-1 RNA at enrolment (copies/mL)**	-	-	-	4.0	3.5–4.6	-	-	-	4.1	3.5–4.4	-	-	-	4.1	3.5–4.7
**NRTI resistance**															
Resistance to both 3TC/FTC and TDF	127	176	72	-	-	46	65	71	-	-	84	117	72	-	-
Resistance to either 3TC/FTC or TDF	40	176	23	-	-	13	65	20	-	-	26	117	22	-	-
No NRTI resistance detected	9	176	5	-	-	6	65	9	-	-	7	117	6	-	-

3TC, lamivudine; ART, antiretroviral therapy; ARTIST, Antiretroviral Therapy in Second-line: investigating Tenofovir-lamivudine-dolutegravir; BMI, body mass index; CD4, cluster of differentiation 4; eGFR, estimated glomerular filtration rate; FTC, emtricitabine; NRTI, nucleoside reverse transcriptase inhibitor; TDF, tenofovir.

† , All values are reported as median (interquartile range) or *n* (%) unless otherwise specified;

‡ , Refers to the pooled cohort from stage one and stage two of the ARTIST trial;

§ , Body Mass Index (BMI) categories: underweight refers to BMI ≤ 18.5 kg/m^2^; normal, BMI 18.5–25 kg/m^2^; overweight, BMI 25–30 kg/m^2^; obese, BMI ≥ 30 kg/m^2^.

### Virologic outcomes

The virologic outcomes during the clinical trial period are displayed in Figure 1, and longer-term (≥ 48 weeks) virologic outcomes, including post-trial follow-up, are presented in Table 2. At 48 weeks, 151/177 (85%; 95% CI 79, 90) of those with HIV-1 RNA results and 151/186 (81%; 95% CI 75, 87) using a mITT analysis had HIV-1 RNA < 50 copies/mL (Table 2 and Online Appendix 1, Table 2-A1). During the clinical trial period, 74% (95% CI 62, 83) of 72 participants at 96 weeks were virologically suppressed, and 78% (95% CI 70, 85) of 127 participants with post-trial follow-up were found to be virologically suppressed (Table 2). Virologic outcomes during post-trial follow-up for the unsuppressed participants were: 14/127 (11%) had HIV-1 RNA 50 copies/mL – 999 copies/mL and 14/127 (11%) had HIV-1 RNA ≥ 1000 copies/mL. When stratified according to the presence of NRTI resistance at enrolment, we found that participants with resistance to both NRTIs at enrolment were more likely to be suppressed at week 48 (92% [95% CI 86, 96]) compared to those with at least one active NRTI (72% [95% CI 57, 84]) (*P* < 0.01), but no significant differences were observed at later time points (Online Appendix 1, Table 3-A1).

**TABLE 2:** Longer-term virologic outcomes in ARTIST trial participants on tenofovir-lamivudine-dolutegravir as second-line antiretroviral therapy.

Timepoint	HIV-1 RNA < 50 copies/mL				HIV-1 RNA ≥ 50 copies/mL				HIV-1 RNA 50–999 copies/mL				HIV-1 RNA ≥ 1000 copies/mL			
	*n*	*N*	%	95% CI	*n*	*N*	%	95% CI	*n*	*N*	%	95% CI	*n*	*N*	%	95% CI
**Follow-up visits during the clinical trial period †**																
Week 48 visit	151‡	177	85	79–90	26	177	15	10–21	17	177	10	6–15	9	177	5	1–6
Week 72 visit	95	114	83	75–90	19	114	17	10–25	14	114	12	7–20	5	114	4	1–9
Week 96 visit	53	72	74	62–83	19	72	26	17–38	11	72	15	8–26	8	72	11	5–21
Week 120 visit	26	34	76	59–89	8	34	24	11–41	4	34	12	3–27	4	34	12	3–27
Week 144 visit	20	29	69	49–85	9	29	31	15–51	3	29	10	2–27	6	29	21	8–40
**Post-trial follow-up visit §**																
All participants¶	99	127	78	70–85	28	127	22	15–30	14	127	11	6–18	14	127	11	6–18
72–96 weeks on TLD	22	27	81	62–94	5	27	19	6–38	2	27	7	1–24	3	27	11	2–29
96–144 weeks on TLD	48	62	77	65–87	14	62	23	13–35	9	62	15	7–26	5	62	8	3–18
≥ Week 144 weeks on TLD	29	38	76	60–89	9	38	24	11–40	3	38	8	2–22	6	38	16	6–31

ART, antiretroviral therapy; ARTIST, Antiretroviral Therapy in Second-line: investigating Tenofovir-lamivudine-dolutegravir; RNA, ribonucleic acid; TLD, tenofovir-lamivudine-dolutegravir.

† , July 2019 to October 2022. Denominator (N) refers only to those with available HIV-1 ribonucleic acid (RNA) results at the indicated visit and within the window period for that visit. Visits were conducted with a ±6-week window period;

‡ , 151/186 (81%; 95% CI 75–87) if a modified intention to treat (mITT) population was used. See Online Appendix 1, Table 2-A1;

§ , Post-trial follow-up conducted from May to September 2023;

¶ , Median (interquartile range) duration since the initiation of tenofovir-lamivudine-dolutegravir was 124 weeks (interquartile range 101 weeks–162 weeks).

The enrolment characteristics of participants who were suppressed at week 96 and post-trial follow-up were similar to unsuppressed participants at these timepoints (Table 3); however, those with a higher enrolment HIV-1 RNA were more likely to be unsuppressed at post-trial follow-up. In unsuppressed participants at week 96 of the clinical trial period or during the post-trial follow-up, we found that 12/16 (75%) and 15/28 (54%), respectively, resuppressed (HIV-1 RNA < 50 copies/mL) at subsequent visits after enhanced adherence counselling.

**TABLE 3:** A comparison by virologic suppression status of enrolment characteristics, nucleoside reverse transcriptase inhibitor resistance and previous episodes of viraemia in participants in the ARTIST trial with longer-term virologic outcome data available.

Variable	Week 96 of clinical trial period (*N* = 72)†													Post-trial visits (*N* = 127)‡												
	HIV-1 RNA < 50 copies/mL (*N* = 53)						HIV-1 RNA ≥ 50 copies/mL (*N* = 19)						*P*	HIV-1 RNA < 50 copies/mL (*N* = 99)						HIV-1 RNA ≥ 50 copies/mL (*N* = 28)						*P*
	*n*	*N*	Median	IQR	%	95% CI	*n*	*N*	Median	IQR	%	95% CI		*n*	*N*	Median	IQR	%	95% CI	*n*	*N*	Median	IQR	%	95% CI	
**Characteristics at TLD initiation**																										
Age	-	-	39	31–46	-	-	-	-	36	29– 39	-	-	0.21	-	-	39	33– 46	-	-	-	-	36	32– 42	-	-	0.30
Sex (female)	40	53	-	-	75	-	16	19	-	-	84	-	0.64	71	99	-	-	72	-	19	28	-	-	68	-	0.83
HIV-1 RNA at enrolment (log_10_ copies/mL)	-	-	3.98	3.42– 4.42	-	-	-	-	4.32	4.00– 4.45	-	-	0.14	-	-	3.92	3.42– 4.65	-	-	-	-	4.31	4.02–4.93	-	-	< 0.01
CD4 count at enrolment (cells/µL)	-	-	282	193–379	-	-	-	-	256	172– 352	-	-	0.50	-	-	246	187– 343	-	-	-	-	243	134– 332	-	-	0.23
**Genotypic antiretroviral resistance testing at TLD initiation**																										
Dual NRTI resistance§	36	49	-	-	73	59–85	10	15	-	-	66	38–88	0.85	63	91	-	-	69	59–78	21	26	-	-	81	61–93	0.36
**Virologic suppression at previous time points**																										
HIV-1 RNA < 50 copies/mL at week 24	51	53	-	-	96	87– 100	15	19	-	-	79	54– 94	0.06	85	99	-	-	88	80–94	24	28	-	-	86	67– 96	1.0
HIV-1 RNA < 50 copies/mL at week 48	46	53	-	-	90	78– 97	12	19	-	-	63	38–84	0.05	86	98	-	-	87	79–94	23	28	-	-	82	63– 94	0.65
**Achieved virologic suppression at subsequent visits after enh anced adherence counselling**																										
HIV-1 RNA < 50 copies/mL	-	-	-	-	-	-	12	16	-	-	75	48–93	-	-	-	-	-	-	-	15	28	-	-	54	34–72	-

ART, antiretroviral therapy; ARTIST, Antiretroviral Therapy in Second-line: investigating Tenofovir-lamivudine-dolutegravir; CD4, cluster of differentiation 4; IQR, interquartile range; NRTI, nucleoside reverse transcriptase inhibitor; RNA, ribonucleic acid; TLD, tenofovir-lamivudine-dolutegravir.

† , July 2019 to October 2022. Denominator refers to those with available HIV-1 ribonucleic acid results at the indicated visit and within the window period for that visit. Visits were conducted with a 6-week window period;

‡ , Post-trial follow-up conducted from May to September 2023. Median (interquartile range) duration since the initiation of tenofovir-lamivudine-dolutegravir was 124 weeks (101–162);

§ , Both tenofovir and lamivudine have a Stanford score ≥15.

**FIGURE 2:**
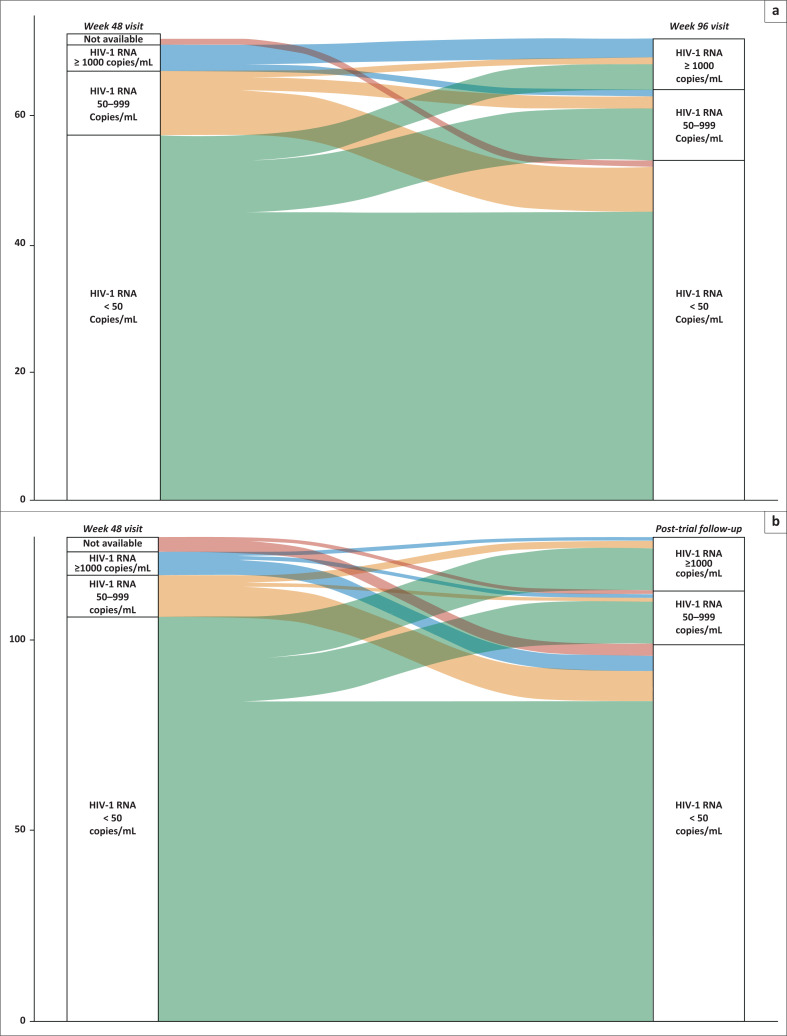
Alluvial diagrams showing transitions between virologic outcomes comparing (a) participants at week 48 and those with outcome data at week 96 of the clinical trial period (*n* = 72); and (b) participants at week 48 and those with outcome data at post-trial follow-up period (*n* = 127).

The week 48 and week 96 virologic outcomes were compared in those with data available, and participants’ categorical transitions between these timepoints are displayed in Figure 2a. We found that 7/14 (50%) participants with unsuppressed HIV-1 RNA at week 48 and available data at week 96 had transitioned into the virologically suppressed category by this visit, and 44/56 (79%) participants who were suppressed at week 48, remained suppressed at week 96. In the post-trial follow-up period, 12/17 (71%) of participants who were unsuppressed at 48 weeks had achieved virologic suppression at the post-trial visit (Figure 2b).

We combined HIV-1 RNA data from samples taken by local HIV clinics with the clinical trial and post-trial follow-up visits and found that longer-term virologic outcomes were similar using combined data from these multiple settings (Online Appendix 1, Table 4-A1). Using these combined data, we found that 137/151 (91%) participants who were suppressed at the week 48 visit had one or more subsequent documented HIV-1 RNA measurement, and 70% of these participants maintained virologic suppression in all available samples taken at subsequent time points. Resistance to both NRTIs was present in higher proportions of participants with virologic rebound and these participants had higher enrolment HIV-1 RNA (Online Appendix 1, Table 5-A1). In those with at least one episode of viraemia after week 48, 28/36 (78%) of those with available data were found to resuppress subsequently to an HIV-1 RNA < 50 copies/mL (Online Appendix 1, Table 5-A1).

### Genotypic antiretroviral resistance testing

Twenty-nine participants met criteria for GART, with three participants meeting criteria for GART on two occasions (Table 4). Two of the 32 (6%) tests conducted failed to amplify and, in one participant, the protease and reverse transcriptase fragment did not amplify but integrase was still tested. The median (IQR) duration on TLD at the time of GART was 95 weeks (49–143) and 21 participants (78%; *N* = 27 available test results) had dual NRTI resistance at enrolment.

**TABLE 4:** HIV-1 Genotypic Antiretroviral Resistance Testing (GART) findings in ARTIST participants with viraemia and meeting criteria for GART.

	*n*	%	Median	IQR
**GART results available (*N* = 32) †**				
Stage 1 and 2	15	47	-	-
Post-trial follow-up	17	53	-	-
**Participant characteristics at enrolment (*N* = 28)**				
Age	-	-	36	32–42
Sex (female)	18	62	-	-
Baseline CD4 lymphocyte count in cells/µL	-	-	176	128–324
Baseline HIV-1 RNA in log_10_ copies/mL	-	-	4.3	4.0–4.9
Baseline dual NRTI resistance (*N* = 27)	21	78	-	-
**Laboratory findings at the time of GART (*N* = 30)**				
CD4 lymphocyte count in cells/µL	-	-	242	144–379
HIV-1 RNA in log_10_ copies/mL	-	-	4.1	2.9–5.2
**Duration from TLD initiation to GART (*N* = 30)**				
Duration in weeks	-	-	95	49–143
≤ 48 weeks	8	27	-	-
48–96 weeks	8	27	-	-
96–144 weeks	6	20	-	-
≥ 144 weeks	8	27	-	-
**NRTI resistance mutations‡ (*N* = 30)**				
0	14	46	-	-
1–2	9	30	-	-
≥ 3	7	23	-	-
**NNRTI resistance mutations§ (*N* = 30)**				
0	3	10	-	-
1–2	16	53	-	-
≥ 3	11	37	-	-
**INI resistance mutations¶ (*N* = 30)**				
0	28	93	-	-
1–2	1	3	-	-
≥ 3	1	3	-	-
**PI resistance mutations (major)| (*N* = 30)**				
0	29	97	-	-
1–2	1	3	-	-
≥ 3	0	0	-	-
**Stanford resistance levels†† (*N* = 30)**				
**Lamivudine resistance levels**			-	-
Susceptible	15	57	-	-
Intermediate resistance	1	3	-	-
High-level resistance	11	37	-	-
Not tested	1	3	-	-
**Tenofovir resistance levels**			-	-
Susceptible	20	67	-	-
Low-level resistance	4	13	-	-
Intermediate resistance	1	3	-	-
High-level resistance	4	13	-	-
Not tested	1	3	-	-
**Dolutegravir resistance levels**			-	-
Susceptible	21	70	-	-
Intermediate resistance	1	3	-	-
High-level resistance	1	3	-	-
Not tested	7	23	-	-

Note: During the clinical trial period, GART was conducted in participants with repeat HIV-1 RNA ≥ 500 copies/mL 2 weeks after enhanced adherence counselling, and in all participants with HIV-1 RNA ≥ 500 copies/mL at the post-trial follow-up. In the post-trial follow-up, GART was omitted if participants reported having interrupted antiretroviral therapy and were not on ART at the time of the visit.

ART, antiretroviral therapy; ARTIST, Antiretroviral Therapy in Second-line: investigating Tenofovir-lamivudine-dolutegravir; CD4, cluster of differentiation 4; GART, genotypic antiretroviral resistance testing; INI, integrase inhibitor; NRTI, nucleoside reverse transcriptase inhibitor; NNRTI, non-nucleoside reverse transcriptase inhibitor; PI, protease inhibitor; TLD, tenofovir-lamivudine-dolutegravir.

† , 32 genotypic antiretroviral resistance testing conducted in 29 participants (three participants had repeated GART at two different time points); two tests were excluded due to failure of amplification in the protease, reverse transcriptase, and integrase gene fragments; in one test there was failure of amplification in protease and reverse transcriptase but not in integrase and this test was included;

‡ , NRTI resistance mutations: *(1) Non-thymidine analogue mutations*: M184I/V (*n* = 11); K65R (*n* = 4); L74I (*n* = 2); Y115F (*n* = 3); K70E/N (*n* = 5); *(2) Thymidine analogue mutations (TAMs)*: K70R (*n* = 1), K219E/R/Q (*n* = 4), T215Y (*n* = 1), D67N (*n* = 4), L210F (*n* = 1), M41L (*n* = 1), A62V (*n* = 1);

§ , NNRTI resistance mutations: *(1) Major*: L100I (*n* = 0), K101E (*n* = 1), K103N/S (*n* = 23), V106M (*n* = 3), Y181C/I/V (*n* = 0), Y188H/Y (*n* = 1), G190A/R/S (*n* = 3), F227 (*n* = 3), M230L (*n* = 1). *(2) Additional NNRTI mutations*: A98G (*n* = 2), E138A/G/Q (*n* = 9);

¶ , INI resistance mutations: *(1) Major*: G118R (*n* = 2), E138K (*n* = 1), T66A (*n* = 1). *(2) Accessory*: G163R (*n* = 1) | PI resistance mutations: *(1) Major*: V82I/V (*n* = 1);

†† , Stanford scoring system: *(1)* ‘Susceptible’, total score < 10; *(2)* ‘Potential low-level resistance’, total score between 10 and 14; *(3)* ‘Low-level resistance’, total score between 15 and 29; *(4)* ‘Intermediate resistance’, total score between 30 and 59; and (v) ‘High-level resistance’, total score ≥ 60.

**FIGURE 3:**
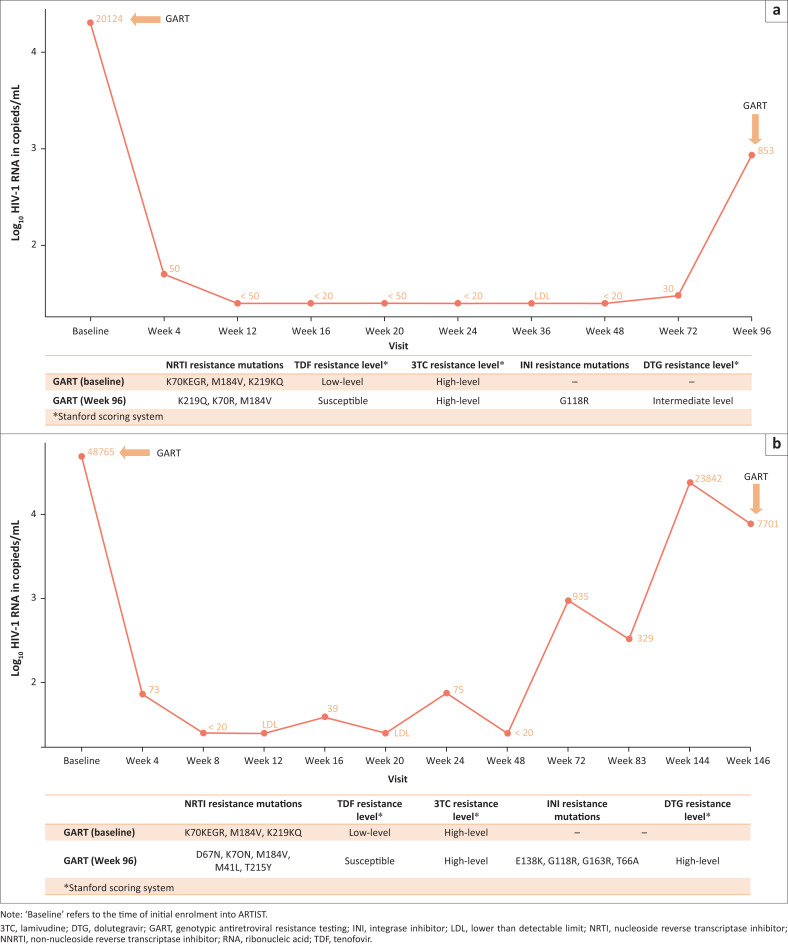
Diagrams depicting the HIV-1 ribonucleic acid (RNA) trajectories in two participants who developed dolutegravir resistance. Participant (a) developed intermediate dolutegravir resistance (Stanford score: 50), detected at week 96; clinical information: female, 40 years old, baseline HIV-1 RNA 4.30 log_10_ copies/mL, baseline CD4 lymphocyte count: 256 cells/μL. Participant (b) developed high-level dolutegravir resistance (Stanford score: 70), detected at week 146; clinical information: Male, 39 years old, baseline HIV-1 RNA 4.30 log_10_ copies/mL, baseline CD4 lymphocyte count: 175 cells/μL.

Dolutegravir resistance was detected in two participants (7%) with at least one GART result (i.e., 1% of the 192 participants in the ARTIST cohort). In one participant, virologic rebound to 853 copies/mL developed at week 96 after being virologically suppressed until week 72 (Figure 3a), and intermediate-level dolutegravir resistance with the G118R mutation was detected at GART. In the other participant, a gradual HIV-1 RNA increase was noted from week 72 to week 144 (Figure 3b), and the participant had an HIV-1 RNA value of 7701 copies/mL with high-level dolutegravir resistance (E138K, G118R, G163R, and T66A mutations) at week 146. In both participants, resistance to both tenofovir and lamivudine in addition to high-level NNRTI resistance was present at the time of initiation of TLD, with the specific DRMs shown in Figure 3a and Figure 3b. Both participants were switched to tenofovir-emtricitabine-darunavir/ritonavir as third-line ART and both were found to resuppress (HIV-1 RNA < 50 copies/mL) by the completion of the post-trial follow-up period.

### Longer-term post-trial clinical outcomes

We considered the longer-term post-trial clinical outcomes including CD4 count, hospitalisation, all-cause mortality, and incident tuberculosis for participants at the post-trial follow-up visits (Online Appendix 1, Table 6-A1). Participants had a median increase (95% CI) in CD4 count of 192 cells/µL (155, 223) since TLD initiation, and the cumulative incidence of tuberculosis was 3/127 (2%). In total, four participants (2%) died during the clinical trial and post-trial period. Two participants died during the clinical trial period, one of COVID-19 pneumonia and the other of sepsis-related complications. At the post-trial follow-up visit, a further two participants had died; one of sepsis and the other from trauma. Hospitalisation was documented in 9/127 (7%) participants, of which three causes for admission were unrelated to HIV.

### Objective measure of adherence

TFV-DP concentrations in dried blood spots were available for 168/177 (95%) participants with available HIV-1 RNA data at week 48. The median (IQR) TFV-DP concentration was 1194 fmol/punch (910, 1653), and was higher in the participants who were virologically suppressed at this time point (1257 fmol/punch; IQR 963, 1694) compared to those who were unsuppressed (842 fmol/punch; IQR 472, 1390) (*P* < 0.001). The relationship between virologic outcomes at week 48 and objective adherence as measured by TFV-DP concentrations in dried blood spots is shown in the Online Appendix 1, Figure 2-A1. The participant who developed dolutegravir resistance at week 96 was found to have a TFV-DP concentration within the lower quartile (681 fmol/punch) at week 48, despite being virologically suppressed at this visit. Conversely, the participant who developed dolutegravir resistance at week 146 did not have a TFV-DP concentration below the median value of the cohort at this timepoint. At post-trial follow-up, lower TFV-DP concentrations were significantly associated with HIV-1 RNA ≥ 1000 copies/mL (161 [IQR 53, 758]) compared to HIV-1 RNA < 50 copies/mL (965 [IQR 761, 1101]) (*P* < 0.01). Additionally, TFV-DP concentrations in DBS confirmed improved adherence to ART from visit one (549, [IQR 163, 1050]) to visit two (895 [IQR 418, 1107]) and visit three (1020 [IQR 779, 1325]), suggesting that enhanced adherence counselling was having a positive effect.

## Discussion

Our longer-term follow-up of ARTIST participants confirms that virologic outcomes for people on TLD as second-line ART are durable and that approximately 80% of participants remain virologically suppressed when follow-up is extended to 3 years. We show that the proportion of participants with virologic suppression at 1 year is comparable at 2 years of follow-up; and that similar proportions of virologic suppression are found in participants being followed up in routine care beyond follow-up in the clinical trial setting. Treatment-emergent dolutegravir resistance was uncommon (~1%) on second-line TLD in this cohort.

The favourable longer-term virologic outcomes found in this study are comparable to other clinical trials that assessed the efficacy of second-line TLD with recycled NRTIs.^3,19,20^ These findings add to the body of evidence supporting the use of TLD in second-line ART and provide reassuring data for many LMICs where second-line TLD is already standard of care.^21^ Virologic suppression at week 48 in our study (~80%) was similar to virologic suppression demonstrated in the VISEND (82% HIV-1 RNA < 1000 copies/L) and D2EFT (78% HIV-1 RNA < 50 copies/L) trials.^20,22^ Similarly, proportions with virologic suppression at 96 weeks (74%) and post-trial follow-up through 158 weeks (78%) were comparable to week 96 results from NADIA, where 189/235 (80%) of the dolutegravir group achieved an HIV-1 RNA < 50 copies/mL at this time point.^3^ In addition, we found that although approximately 30% of participants who are virologically suppressed at 1 year on second-line TLD will have subsequent episodes of viraemia, the majority resuppressed after enhanced adherence counselling. This is consistent with our previous findings, where we showed that the trajectory of most ARTIST participants with transient viraemia was to resuppressed, thus highlighting the importance of adherence support and virologic monitoring in those on TLD as second-line therapy.^12^

We found that participants with resistance to both NRTIs on enrolment genotypic resistance testing were more likely to be virologically suppressed at 48 weeks of follow-up. The absence of NRTI resistance at the time of changing regimens often implies poor adherence, which likely persists on second-line ART, and may explain this finding. Additionally, the cost to viral fitness with NRTI resistance and, in particular, the crippling nature of the M184V/I and the K65R/N mutations, contributes to the efficacy of TLD in the absence of a fully active NRTI.^23^ In NADIA, more than 90% of participants with no predicted active NRTIs achieved HIV-1 RNA < 400 copies/mL at week 48, and, in the subgroup with the K65R/N mutation, 96% achieved suppression in the tenofovir group.^19,24^ Moreover, the nucleoside resistance mutation, K65R, has been shown to prevent the emergence of dolutegravir resistance in vitro.^25^ These factors may highlight the advantage of using of TLD as second-line ART and the recycling of tenofovir and lamivudine/emtricitabine in third-line ART for those failing TLD.^4,26,27^ However, conversely, a large observational study showed that NRTI resistance was an important risk factor for the development of dolutegravir resistance.^10^

We detected acquired dolutegravir resistance in two participants (~1% of this cohort) and dolutegravir resistance was detected at durations of 96 and 146 weeks after the initiation of TLD as second-line ART. This is consistent with other trial and programmatic data where dolutegravir resistance has been reported in 2% – 4% of patients on dolutegravir-based second-line ART.^2,3,6,8^ In our cohort, both cases of dolutegravir resistance were detected after 72 weeks duration of TLD and it is possible that, because of dolutegravir’s high genetic barrier to resistance, higher proportions of emergent resistance may only be detected after longer durations of second-line dolutegravir-based therapy.^28^ It is also possible that dolutegravir resistance could develop more rapidly in settings where individuals have unchecked viraemia for significant periods of time. In DAWNING (a phase 3b trial comparing dolutegravir to LPV/r, both with dual NRTI therapy, in adults failing first-line therapy), two of 312 (1%) participants developed dolutegravir resistance by 48 weeks and, when follow-up was extended to 158 weeks, a further five participants were found to have INSTI resistance mutations.^6,9,29^ In both DAWNING and NADIA, no protease inhibitor (PI) resistance was detected, indicating that the genetic barrier to resistance of dolutegravir is not as high as PIs, the previous standard of care in second-line.^3,6,9,29^

The two participants with dolutegravir resistance in this cohort were found to have resistance to both tenofovir and lamivudine as well as high-level NNRTI resistance at enrolment. A recent analysis of over 700 samples sent for genotypic resistance testing showed that NRTI resistance was a major risk factor for the development of dolutegravir resistance (adjusted odds ratio of 4.62; 95% CI 1.24, 17.2 for potential-low/low, and 7.01; 95% CI 2.52, 19.48 for intermediate/high-level NRTI resistance), which highlights that dolutegravir’s barrier to resistance is significantly lower in second-line compared to first-line therapy where accompanying NRTIs are fully active, despite the fact that the majority of patients achieve virologic suppression on second-line TLD.^10^ These findings together suggest that although dual NRTI resistance does not increase the risk of virologic failure on second-line TLD, it may increase the risk of resistance in those with viraemia.^10^ Other contributing factors which may have increased the risk of dolutegravir resistance developing in these two cases are high-level NNRTI resistance at the time of TLD initiation, sub-optimal adherence, relatively high HIV-1 RNA at TLD initiation (compared to the rest of the cohort), and relatively low CD4 lymphocyte counts (< 300 cells/µL in both cases) (Figure 3a and Figure 3b).^10,29,30,28,31^

### Strengths and limitations

Our study has the following strengths. First, we add to the current literature by reporting some of the longest duration of follow-up data from participants on second-line TLD with recycled NRTIs. Second, we assess virologic outcomes in both a clinical trial setting and a routine-care setting. Third, given the rigorous implementation of our resistance testing indications, it is unlikely that dolutegravir resistance was missed among those participants who attended study visits.

Our study also has limitations. The included sample of participants was relatively small, single-community-based, female-predominant, and had relatively low HIV-1 RNA values at study entry. In addition, missing data at later time points – which was only partially compensated for by using data from the post-trial visits and routine care – is a limitation of this study. However, it is notable that within our setting, the observed loss of contact is more likely explained by changed contact numbers than genuine loss to follow-up from routine care.

## Conclusion

Our data are reassuring, showing that the longer-term virologic outcomes on TLD as second-line ART are favourable, with sustained virologic suppression in approximately 70% – 80% up to 3 years after the initiation of TLD. This supports current recommendations that tenofovir and lamivudine can be recycled safely and effectively with dolutegravir without the requirement of a genotypic resistance test prior to starting, which has particularly advantageous public health implications for LMICs. Of concern is that a small proportion develop dolutegravir resistance. There is a need for monitoring and management algorithms to be developed for patients on second-line TLD, including reliable objective measures of adherence,^32^ to ensure that dolutegravir resistance is detected timeously in the minority of patients who develop it while avoiding unnecessary costly resistance testing.

## References

[1] World Health Organization. Update of recommended first- and second-line antiretroviral regimens [homepage on the Internet]. 2019 [cited 2023 Sep 21]. Available from: http://apps.who.int/bookorders

[2] Brown D, Kaplan R, Losso M, et al. Efficacy of second-line dolutegravir plus 2 nucleoside reverse transcriptase inhibitors by baseline nucleoside reverse transcriptase inhibitor resistance and nucleoside reverse transcriptase inhibitor use in the DAWNING study. Antivir Ther. 2022;27(1):135965352210774. 10.1177/13596535221077487

[3] Paton NI, Musaazi J, Kityo C, et al. Efficacy and safety of dolutegravir or darunavir in combination with lamivudine plus either zidovudine or tenofovir for second-line treatment of HIV infection (NADIA): Week 96 results from a prospective, multicentre, open-label, factorial, randomised, non-inferiority trial. Lancet HIV. 2022;9(6):e381–e393. 10.1016/S2352-3018(22)00092-3 35460601

[4] Nel J, Meintjes G, Osih R. Southern African HIV Clinicians Society guidelines for antiretroviral therapy in adults: 2023 update. Cape Town: Southern African HIV Clinicians Society; 2023. 10.4102/sajhivmed.v24i1.1528 PMC10546902 37795427

[5] National Department of Health. 2023 ART clinical guidelines for the management of HIV in adults, pregnancy and breastfeeding, adolescents, children, infants and neonates. Pretoria: Department of Health; 2023.

[6] Underwood M, Horton J, Nangle K, et al. Integrase inhibitor resistance mechanisms and structural characteristics in antiretroviral therapy-experienced, integrase inhibitor-naive adults with HIV-1 infection treated with dolutegravir plus two nucleoside reverse transcriptase inhibitors in the DAWNING study. Antimicrob Agents Chemother. 2022;66(1):e01643-21. 10.1128/AAC.01643-21 34694877 PMC8765460

[7] Wijting I, Rokx C, Boucher C, et al. Switching from cART to dolutegravir (DTG) maintenance monotherapy in virologically suppressed HIV-1 infected adults: A randomized multicenter, non-inferiority clinical trial (DOMONO). J Int AIDS Soc. 2016;19(suppl. 7):0334.

[8] Schramm B, Temfack E, Descamps D, et al. Viral suppression and HIV-1 drug resistance 1 year after pragmatic transitioning to dolutegravir first-line therapy in Malawi: A prospective cohort study. Lancet HIV. 2022;9(8):e544–e553. 10.1016/S2352-3018(22)00136-9 35905753

[9] Aboud M, Kaplan R, Lombaard J, et al. Dolutegravir versus ritonavir-boosted lopinavir both with dual nucleoside reverse transcriptase inhibitor therapy in adults with HIV-1 infection in whom first-line therapy has failed (DAWNING): An open-label, non-inferiority, phase 3b trial. Lancet Infect Dis. 2019;19(3):253–264. 10.1016/S1473-3099(19)30036-2 30732940

[10] Loosli T, Hossmann S, Ingle SM, et al. HIV-1 drug resistance in people on dolutegravir-based antiretroviral therapy: A collaborative cohort analysis. Lancet HIV. 2023;10(11):e733–e741. 10.1016/S2352-3018(23)00228-X 37832567 PMC10913014

[11] Zhao Y, Griesel R, Omar Z, et al. Initial supplementary dose of dolutegravir in second-line antiretroviral therapy: A noncomparative, double-blind, randomized placebo-controlled trial. Clin Infect Dis. 2023;76(10):1832–1840. 10.1093/cid/ciad023 36645792 PMC10209436

[12] Keene CM, Cassidy T, Zhao Y, et al. Recycling tenofovir in second-line antiretroviral treatment with dolutegravir: Outcomes and viral load trajectories to 72 weeks. J Acquir Immune Defic Syndr. 2023;92(5):422–429. 10.1097/QAI.0000000000003157 36706364 PMC7614301

[13] Keene CM, Griesel R, Zhao Y, et al. Virologic efficacy of tenofovir, lamivudine and dolutegravir as second-line antiretroviral therapy in adults failing a tenofovir-based first-line regimen. AIDS. 2021;35(9):1423–1432. 10.1097/QAD.0000000000002936 33973876 PMC7612028

[14] Zhao Y, Keene C, Griesel R, et al. AntiRetroviral Therapy In Second-line: Investigating Tenofovir-lamivudine-dolutegravir (ARTIST): Protocol for a randomised controlled trial. Wellcome Open Res. 2021;6:33. 10.12688/wellcomeopenres.16597.1 36017341 PMC9372637

[15] Strategic Development Information and GIS Department. City of Cape Town – 2011 Census Suburb Khayelitsha. Cape Town: City of Cape Town; 2013.

[16] Stranix-Chibanda L, Anderson PL, Kacanek D, et al. Tenofovir diphosphate concentrations in dried blood spots from pregnant and postpartum adolescent and young women receiving daily observed pre-exposure prophylaxis in sub-Saharan Africa. Clin Infect Dis. 2021;73(7):e1893–e1900. 10.1093/cid/ciaa1872 33341883 PMC8492211

[17] Asjad Naqvi, 2022. ALLUVIAL: Stata module for Alluvial graphs. Statistical Software Components S459153. Boston: Boston College Department of Economics; 2024.

[18] Brunson J. ggalluvial: Layered grammar for alluvial plots. J Open Source Softw. 2020;5(49):2017. 10.21105/joss.02017 36919162 PMC10010671

[19] Paton NI, Musaazi J, Kityo C, et al. Dolutegravir or darunavir in combination with zidovudine or tenofovir to treat HIV. N Engl J Med. 2021;385(4):330–341. 10.1056/NEJMoa2101609 34289276

[20] Mulenga LB, Fwoloshi S, Mweemba A, et al. Dolutegravir with recycled NRTIs is noninferior to PI-based art: VISEND trial. Top Antivir Med. 2022;30(1 suppl):135.

[21] Steegen K, Hans L. Compelling evidence for unconditional shift to dolutegravir. Lancet HIV. 2022;9(8):e523–e524. 10.1016/S2352-3018(22)00164-3 35905750

[22] Clayden P. CROI 2023: Yet more evidence for recycled tenofovir and 3TC or FTC with dolutegravir in second-line: 48 week results from the D2EFT study [home page on the Internet]. HIV i-Base. 2023 [cited 2023 Sep 11]. Available from: https://i-base.info/htb/44850

[23] Ross L, Parkin N, Lanier R. The number of HIV major NRTI mutations correlates directly with other antiretroviral-associated mutations and indirectly with replicative capacity and reduced drug susceptibility. AIDS Res Hum Retroviruses. 2008;24(4):617–620. 10.1089/aid.2007.0188 18366310

[24] Clayden P. CROI 2022: Dolutegravir plus recycled tenofovir rather than switch to AZT: Public health approach to second-line ART [homepage on the Internet]. HIV i-Base. 2022 [cited 2023 Sep 11]. Available from: https://i-base.info/htb/42725

[25] Oliveira M, Ibanescu RI, Pham HT, Brenner B, Mesplede T, Wainberg MA. The M184I/V and K65R nucleoside resistance mutations in HIV-1 prevent the emergence of resistance mutations against dolutegravir. AIDS. 2016;30(15):2267–2273. 10.1097/QAD.0000000000001191 27367488

[26] Fokam J, Chenwi CA, Takou D, et al. Laboratory based surveillance of HIV-1 acquired drug resistance in Cameroon: Implications for use of tenofovir-lamivudine-dolutegravir (TLD) as second- or third-line regimens. Viruses. 2023;15(8):1683. 10.3390/v15081683 37632026 PMC10459610

[27] Bossard C, Schramm B, Wanjala S, et al. High prevalence of NRTI and NNRTI drug resistance among ART-experienced, hospitalized inpatients. J Acquir Immune Defic Syndr. 2021;87(3):883–888. 10.1097/QAI.0000000000002689 33852504 PMC8191469

[28] Gil H, Delgado E, Benito S, Moreno-Lorenzo M, Thomson MM. Factors associated with HIV-1 resistance to integrase strand transfer inhibitors in Spain: Implications for dolutegravir-containing regimens. Front Microbiol. 2022;13:1051096. 10.3389/fmicb.2022.1051096 36578581 PMC9792149

[29] Cevik M, Orkin C, Sax PE. Emergent resistance to dolutegravir among instinaive patients on first-line or second-line antiretroviral therapy: A review of published cases. Open Forum Infect Dis. 2020;7(6):ofaa202. 10.1093/ofid/ofaa202 32587877 PMC7304932

[30] Kouamou V, Inzaule S, Manasa J. Dolutegravir drug-resistance monitoring in Africa. Lancet HIV. 2021;8(11):e664–e666. 10.1016/S2352-3018(21)00268-X 34735801

[31] Van Oosterhout JJ, Chipungu C, Nkhoma L, et al. Dolutegravir resistance in Malawi’s national HIV treatment program. Open Forum Infect Dis. 2022 Apr 5;9(5):ofac148. 10.1093/ofid/ofac148 35493118 PMC9045949

[32] Orrell C, Cohen K, Leisegang R, Bangsberg DR, Wood R, Maartens G. Comparison of six methods to estimate adherence in an ART-naïve cohort in a resource-poor setting: Which best predicts virological and resistance outcomes? AIDS Res Ther. 2017;14(1):20. 10.1186/s12981-017-0138-y 28376815 PMC5379739

